# Primary Adrenal Insufficiency Masked by an Eating Disorder Diagnosis in an Adolescent Male

**DOI:** 10.1210/jcemcr/luad095

**Published:** 2023-08-09

**Authors:** Presley Nichols, Virginia Rahming, Alyson Weiner, Aviva B Sopher

**Affiliations:** Division of Endocrinology Diabetes and Metabolism, Department of Pediatrics, Columbia University Irving Medical Center and NewYork Presbyterian Morgan Stanley Children's Hospital, New York, NY 10032, USA; Division of Endocrinology Diabetes and Metabolism, Department of Pediatrics, Columbia University Irving Medical Center and NewYork Presbyterian Morgan Stanley Children's Hospital, New York, NY 10032, USA; Division of Endocrinology Diabetes and Metabolism, Department of Pediatrics, Columbia University Irving Medical Center and NewYork Presbyterian Morgan Stanley Children's Hospital, New York, NY 10032, USA; Division of Endocrinology Diabetes and Metabolism, Department of Pediatrics, Columbia University Irving Medical Center and NewYork Presbyterian Morgan Stanley Children's Hospital, New York, NY 10032, USA

**Keywords:** adrenal insufficiency, eating disorder, anchoring bias, case report

## Abstract

We describe a 14-year-old male who was followed for several years for the diagnoses of avoidant restrictive food intake disorder and generalized anxiety disorder before being diagnosed with primary adrenal insufficiency (PAI) or Addison disease. The patient presented multiple times to different facilities with worsening symptoms of anorexia, nausea, vomiting, and anxiety in the months leading up to diagnosis of PAI. Dehydration and hypotension, occurring relatively late in the course of his illness, were attributed to poor intake and vomiting. Hyponatremia was attributed to his psychotropic medication, olanzapine, and to dehydration. During his third hospitalization, he was diagnosed with PAI; treatment with stress-dose glucocorticoid therapy resulted in rapid clinical improvement. This case serves as a reminder that adrenal insufficiency must be considered in the differential diagnosis of eating disorders because signs and symptoms of adrenal insufficiency can overlap and progress insidiously. Additionally, we recognize that the diagnostic process is intertwined with a patient's medical history and use this opportunity to discuss cognitive, specifically anchoring, bias in academic medicine.

## Introduction

Primary adrenal insufficiency (PAI) is characterized by abnormalities of steroid biosynthesis resulting in inadequate production of adrenal glucocorticoids and mineralocorticoids. The initial description in 1855 by Dr. Thomas Addison was in a series of 6 patients with tubercular infiltration of the adrenal gland [1]. PAI is exceedingly rare in both children and adults, with a prevalence of 100 to 140 cases per million [2]. In adults, the most common etiology of PAI is autoimmune destruction of the adrenal gland and, in children, it is congenital adrenal hyperplasia resulting from 21-hydroxylase deficiency. In a recent multicenter cohort study of children with PAI, the second most common cause was X-linked adrenoleukodystrophy, followed by autoimmune polyglandular syndrome type I and isolated autoimmune adrenalitis [3]. Additional etiologies of PAI are listed in Table 1.

**Table 1.** Etiologies of primary adrenal insufficiency

Causes of primary adrenal insufficiency
Autoimmune adrenalitis
Adrenoleukodystrophies
Glandular infiltration by infection: tuberculosis, fungal, cytomegalovirus, HIV
Glandular infiltration by amyloidosis or hemochromatosis
Metastatic neoplasia
Intraadrenal hemorrhage (Waterhouse-Friderichsen syndrome)
Bilateral adrenalectomy
ACTH resistance syndromes: *MC2R*, *MRAP*, or *AAAS* mutations
Congenital adrenal hyperplasia: *CYP21A2* mutation in 90%-95% of cases
Congenital adrenal hypoplasia: *DAX1* or *SF1* mutations

The clinical presentation of PAI in children who do not have classical congenital adrenal hyperplasia is often subtle, with vague symptomatology including fatigue, anorexia, weight loss, and anxiety. These nonspecific symptoms frequently lead to misdiagnosis [4, 5]. We present the case of an adolescent male diagnosed with anxiety and eating disorders 3 years before being diagnosed with autoimmune adrenal insufficiency. This case report serves as a review of a rare but treatable diagnosis and of cognitive biases that drive medical decision making [6, 7].

## Case Presentation

**Figure 1.**
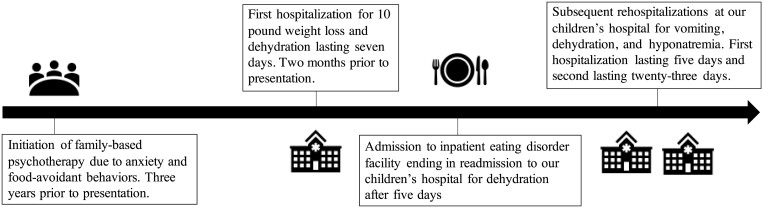
Timeline of diagnostic process and facility stays in the 3 years before diagnosis with autoimmune adrenalitis.

The patient is a White male of English descent with an unremarkable family history who was aged 14 years and 8 months old at the time of diagnosis of PAI. He was diagnosed with anxiety and restrictive eating at age 11 years, which was managed with outpatient psychotherapy. Sertraline was started for worsening anxiety 10 months before diagnosis of PAI. Mirtazapine was added to his regimen for the indication of anxiety and depression with a desired side effect of increased appetite and weight gain when anxiety, nausea, and vomiting became markedly worse 4 months before his diagnosis of PAI. During this period, he had 3 inpatient hospitalizations and 1 admission to an inpatient eating disorder unit (Fig. 1).

The patient was first hospitalized at age 14 years and 4 months for worsening anxiety, anorexia, dehydration, and a 10-pound weight loss. He was treated with isotonic fluid resuscitation followed by nasogastric tube feeds according to the hospital eating disorder protocol. After 2 days, he developed protracted emesis leading to admission to an inpatient eating disorder unit. Thyroid function studies obtained in the medical evaluation for psychiatric conditions were noted to be abnormal, with mild elevation of TSH, low free thyroxine, low total thyroxine, positive antithyroglobulin and antithyroperoxidase antibodies. During this admission, his serum sodium levels ranged from 134 to 140 mmol/L (reference range, 137–145 mmol/L).

Following 5 days at a behavioral health facility, the patient was brought to the emergency department of our children's hospital for management of recurrent dehydration. His serum sodium on presentation was 129 mmol/L. He was admitted to the pediatric hospitalist medicine service with child psychiatry consultation. Because of concern for treatment failure, mirtazapine was discontinued and olanzapine was started on hospital day 1. After 7 days, he was discharged with improved oral intake and stable but persistent hyponatremia, with a sodium level of 134 mmol/L. Hyponatremia was attributed to syndrome of inappropriate antidiuretic hormone related to treatment with olanzapine [8].

**Figure 2.**
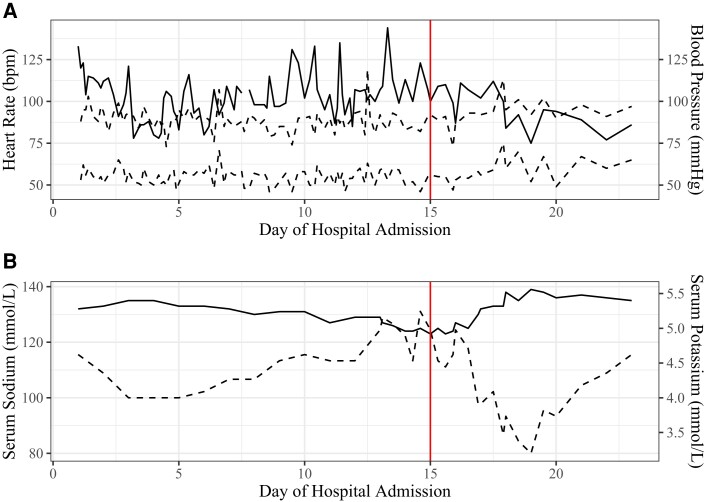
(A) Heart rate and blood pressure trends throughout the course of the third hospitalization. (B) Serum sodium and potassium trends throughout the course of the third hospitalization. Vertical red line in both panels represents the date of hydrocortisone initiation.

Two days following discharge, the patient was again admitted to our children's hospital on the pediatric hospitalist service with adolescent medicine and child psychiatry consultation following presentation to the emergency department for recurrence of vomiting and a presyncopal event. His physical examination showed evidence of dehydration. His heart rate and blood pressure on admission and throughout this hospitalization are shown in Fig. 2A. On admission, his serum sodium was 132 mmol/L, potassium 5.2 mmol/L (reference range, 3.5–5.1 mmol/L), blood urea nitrogen 34 mg/dL or 12.14 mmol/L (reference range, 7–18 mg/dL, 2.5–6.43 mmol/L), and creatinine 1.16 mg/dL or 102.57 µmol/L (reference range, 0.60–1.20 mg/dL, 53.05–106.1 µmol/L). Blood urea nitrogen and creatinine had doubled from 2 days prior. After initial fluid resuscitation, the eating disorder protocol was again followed and the patient was placed on nasogastric tube feeds with close monitoring for refeeding syndrome. Initial electrocardiogram showed a prolonged QTc interval of 499 ms (normal <450 ms). Olanzapine was discontinued because of both prolonged QTc interval and hyponatremia.

Serum sodium and potassium trends throughout this hospitalization are shown in Fig. 2B. An evaluation of hyponatremia revealed elevated urine sodium levels of 154 mEq/L (reference range, <40 mEq/L), urine osmolality of 879 mOsm/kg (reference range, <100 mOsm/kg), and serum osmolality of 271 mOsm/kg (reference range, >280 mOsm/kg) that, together, were consistent with both syndrome of inappropriate antidiuretic hormone and salt wasting. Interpretation of these studies was complicated by ongoing fluid resuscitation with normal saline. On hospital day 11, treatment with enteral salt supplementation was initiated.

## Diagnostic Assessment

In light of hyponatremia, dehydration, salt wasting, and orthostatic hypotension, pediatric endocrinology was consulted and an evaluation for glucocorticoid and mineralocorticoid deficiency was initiated on hospital day 13. His physical examination at that time was notable for lslightly bronzed skin and sexual maturity rating IV pubic hair and external genitalia. The workup documented a low morning cortisol (2.44 µg/dL at 8:23 Am, reference range, 4.8–19.5 µg/dL; 67.32 nmol/L, reference range, 132.43–538.01 nmol/L). A cosyntropin stimulation test performed the following day confirmed the diagnosis of adrenal insufficiency (Table 2). An ACTH level was markedly elevated, confirming the diagnosis of PAI. Very long chain fatty acids were normal and 21-hydroxylase antibodies were positive.

**Table 2.** Laboratory values obtained in the work up of central vs primary adrenal insufficiency and of autoimmune conditions associated with APS

Laboratory parameter	Conventional	SI values
	values (normal range)	(Normal range)
Hemoglobin A1c	5.5 (<5.7%)	0.06 (0.05–0.07 fraction)
Renin	15.84 (0.25–5.82 ng/mL/h)	15.84 (0.25–5.82 µg/h/L)
Aldosterone	4.2 (4.0–31.0 ng/dL)	116.51 (110.96–859.94 pmol/L)
ACTH	1832 (7.2–63.3 pg/mL)	403.41 (1.59–13.94 pmol/L)
Cortisol 0 minutes	2.25 (>5 µg/dL)	62.08 (>137.95 nmol/L)
Cortisol 60 minutes	2.31 (>18 µg/dL)	63.73 (>496.62 nmol/L)
LH	3.4 (1.7–8.6 mIU/mL)	
FSH	8.6 (1.5–12.4 mIU/mL)	
Testosterone	416 (40.1–778.0 ng/dL)	14.42 (1.39–26.97 nmol/L)
PTH	22 (15.1–85.7 pg/mL)	22 (15.1–85.7 ng/L)
Calcium	10.5 (8.8–10.3 mg/dL)	2.62 (2.2–2.57 mmol/L)
Parietal cell Ab, IgG	2.3 (0.0–24.9 U)	
TSH	7.68 (0.41–4.81 mIU/L)	
Free thyroxine	1.39 (0.83–1.9 ng/dL)	17.89 (10.68–24.46 pmol/L)
Thyroid peroxidase Ab	164.18 (<5.61 IU/mL)	
Thyroglobulin Ab	74.82 (<4.11 IU/mL)	
Tissue transglutaminase Ab	<2 (0–3 U/mL)	
IgA, serum	126 (47.0–249.0 mg/dL)	7.86 (2.94–15.56 µmol/L)
GAD-65 Ab	0 (<0.02 nmol/L)	
Insulin Ab	<0.4 (0.0–0.4 U/mL)	
Islet cell Ab, IgG, titer	<1:4 (<1:4)	
21-Hydroxylase Ab	Positive (negative)	

Abbreviations: Ab, antibody; APS, autoimmune polyglandular syndrome; GAD, glutamic acid decarboxylase

The combination of positive 21-hydroxylase antibodies and positive thyroid antibodies in this patient led to the consideration of autoimmune polyglandular syndrome. Further studies were obtained to evaluate for other autoimmune conditions including celiac disease, insulin-dependent diabetes mellitus, pernicious anemia, gonadal failure, and hypoparathyroidism (Table 2). An initial aldosterone level obtained while the patient was hyponatremic was inappropriately normal, with appropriately elevated plasma renin activity. Mineralocorticoid deficiency was confirmed 3 weeks after discharge from the hospital when a repeated aldosterone level was low with elevated renin.

## Treatment

Stress-dose glucocorticoid therapy with hydrocortisone was initiated on hospital day 15 following abnormal cosyntropin stimulation response. Within days of initiating hydrocortisone therapy, the patient's nausea, anxiety, and appetite improved, as did his vital signs and laboratory abnormalities. Treatment with fludrocortisone was initiated 3 weeks after hospital discharge following confirmation of mineralocorticoid deficiency and elevated serum renin while eunatremic. Treatment with levothyroxine was also initiated 3 weeks after hospital discharge in the setting of known thyroid autoantibodies and persistent mild TSH elevation with low-normal free thyroxine.

## Outcome and Follow-up

**Figure 3.**
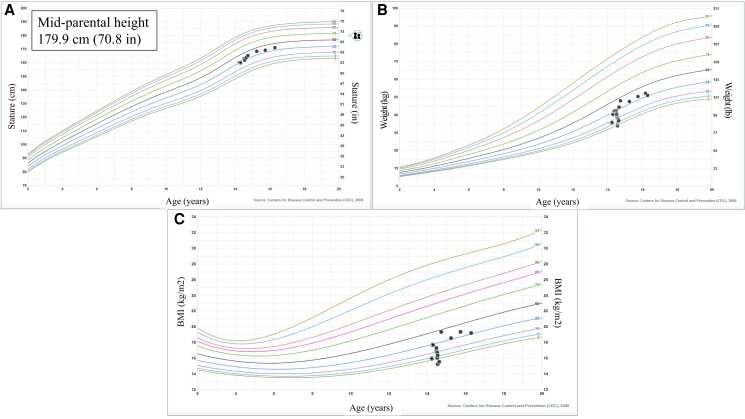
The described patient's (A) height, (B) weight, and (C) body mass index curve on Centers for Disease Control and Prevention male 2-20 years growth curves.

Presently, the patient is being treated with hydrocortisone 18 mg/m^2^/day, fludrocortisone 0.15 mg daily, and levothyroxine 25 mcg daily. His anxiety and appetite have markedly improved and electrolyte levels have remained normal on his current medication regimen. Since discharge, the patient has gained 24 pounds with improvement in body mass index from the 0.78th percentile to the 46th percentile (Fig. 3).

## Discussion

In this 14-year, 8-month-old adolescent male with chronic anorexia, nausea, vomiting, and weight loss, PAI resulting from autoimmune adrenalitis was diagnosed during a third consecutive hospital admission for dehydration. The delay in diagnosing PAI in this patient was largely related to his anchoring psychiatric diagnosis. It is important to recognize cognitive biases because they can contribute to medical error, outcome disparities, and higher health care costs[7, 9]. Anchoring bias is a type of cognitive bias in which a health care provider prematurely arrives to a conclusion to which future decision-making is “anchored.” In the case presented here, the diagnosis of PAI was likely not thoroughly considered during the first or second hospitalization despite findings of hyponatremia and even a comorbid autoimmune condition because of the anchor of being hospitalized for an eating disorder. An earlier and broader consideration of his symptoms of nausea and vomiting with clinical dehydration and hyponatremia despite the interventions of medication adjustment and fluid resuscitation may have prompted an earlier evaluation for PAI, allowing the patient and his family to avoid expensive and psychosocially taxing repeated hospitalizations. Our motivation for reviewing this case and sharing the experience broadly is to increase awareness of overlapping symptoms of adrenal insufficiency and eating disorders, and to highlight the importance of a collaborative approach to the management of challenging cases.

Though pediatric PAI is rare, this patient's symptoms of fatigue, anorexia, and nausea progressing to vomiting, dehydration, hyponatremia, and hyperkalemia are a typical presentation of this disease. In autoimmune adrenalitis, cooccurrence of glucocorticoid and mineralocorticoid deficiencies is common. Mineralocorticoid deficiency results in the classic electrolyte derangements of hyponatremia and hyperkalemia and subsequent profound dehydration.

Once hypocortisolism is identified as the underlying cause of symptoms, it is important to distinguish between secondary or central (ACTH deficiency from the pituitary gland) and primary (inadequate production of cortisol from the adrenal glands) PAI. This is accomplished by obtaining an ACTH level before initiation of glucocorticoid replacement therapy. In this case, ACTH levels were elevated when cortisol was low, suggesting an appropriate pituitary response. The most likely etiologies of PAI in an adolescent male are autoimmune destruction of the adrenal gland and X-linked adrenoleukodystrophy; therefore, 21-hydroxylase antibodies and very long chain fatty acids are the next tests obtained in a step-wise approach to PAI evaluation.

Autoimmune adrenalitis can occur alone or with additional autoimmune pathology, referred to as autoimmune polyglandular syndromes (APS). There are 3 autoimmune polyglandular syndromes; however, only APS 1 and 2 include PAI. APS 1 consists of PAI, hypoparathyroidism, and chronic mucocutaneous candidiasis with 2 of 3 required for diagnosis. APS 1 is also referred to as APECED syndrome (autoimmune polyendocrinopathy, candidiasis ectodermal dystrophy) and can also include primary gonadal failure, Hashimoto hypothyroidism, pernicious anemia, autoimmune hepatitis, type 1 diabetes mellitus, and ectodermal dystrophy. APS 1 is secondary to a mutation in the *AIRE* (autoimmune regulator) gene. APS 2 consists of PAI, Hashimoto hypothyroidism, and type 1 diabetes. Although these are the most common findings, additional autoimmune diseases can include celiac disease, vitiligo, alopecia, myasthenia gravis, pernicious anemia, hepatitis, and hypogonadism. APS 2 is secondary to mutations within the HLA complex.

The case of this adolescent male who presented with anorexia, vomiting, and dehydration exemplifies cognitive bias that delayed diagnosis and administration of treatment. Although it can be helpful for physicians to have “illness scripts” in mind, it is essential to reconsider a diagnosis when a patient does not respond as expected or deteriorates despite medical interventions.

## Learning Points

- The 3 most common causes of primary adrenal insufficiency in children are congenital adrenal hyperplasia, X-linked adrenoleukodystrophy, and autoimmune adrenalitis.
- Primary adrenal insufficiency resulting from autoimmune adrenalitis can have a subtle presentation with features that overlap with generalized anxiety disorder and anorexia nervosa.
- When multiple hormonal pathways are disrupted, is important to consider the order of treatment initiation. In this case, it is important to replace hydrocortisone before levothyroxine to avoid exacerbation of adrenal insufficiency.

## Data Availability

Data sharing is not applicable to this article as no datasets were generated or analyzed during the current study.

## Abbreviations

APS: autoimmune polyglandular syndrome
PAI: primary adrenal insufficiency
